# Fainting Fanconi syndrome clarified by proxy: a case report

**DOI:** 10.1186/s12882-017-0649-8

**Published:** 2017-07-11

**Authors:** Stephen Benedict Walsh, Robert Unwin, Robert Kleta, William van’t Hoff, Paul Bass, Khalid Hussain, Sian Ellard, Detlef Bockenhauer

**Affiliations:** 1 Great Ormond Street Hospital for Children NHS Foundation Trust, London, UK; 2 UCL Centre for Nephrology, Renal Unit, Great Ormond Street Hospital for Children NHS Foundation Trust, Great Ormond Street, London, WC1N 3JH UK; 3 Sidra Medical Centre, Doha, Qatar; 4 University of Exeter Medical School, Institute of Biomedical and Clinical Science, Exeter, UK

**Keywords:** Renal Fanconi syndrome, Hyperinsulinism, Hypoglycaemia, HNF4A, Mitochondrial cytopathy, Genetic testing

## Abstract

**Background:**

Rare diseases may elude diagnosis due to unfamiliarity of the treating physicians with the specific disorder. Yet, advances in genetics have tremendously enhanced our ability to establish specific and sometimes surprising diagnoses.

**Case presentation:**

We report a case of renal Fanconi syndrome associated with intermittent hypoglycemic episodes, the specific cause for which remained elusive for over 30 years, despite numerous investigations, including three kidney and one liver biopsy. The most recent kidney biopsy showed dysmorphic mitochondria, suggesting a mitochondrial disorder. When her son presented with hypoglycemia in the neonatal period, he underwent routine genetic testing for hyperinsulinemic hypoglycemia, which revealed a specific mutation in HNF4A. Subsequent testing of the mother confirmed the diagnosis also in her.

**Conclusion:**

Modern sequencing technologies that test multiple genes simultaneously enable specific diagnoses, even if the underlying disorder was not clinically suspected. The finding of mitochondrial dysmorphology provides a potential clue for the mechanism, by which the identified mutation causes renal Fanconi syndrome.

## Background

There is a very large number of rare diseases, which most clinicians will never encounter in their lifetime, posing a challenge to the establishment of a correct diagnosis. As of November 1st 2016, there were 8270 different phenotypic descriptions of diseases listed in the Online Mendelian Inheritance in Man database (OMIM, http://www.omim.org). Since the completion of the Human Genome Project in 2003, our understanding of the genetic cause of these disease has improved dramatically and with it our ability to make precise diagnoses [1]. Currently, just over half (4865) of the phenotypes listed in OMIM have an identified molecular basis and that number is increasing rapidly. This brings about a shift in the practice of medicine such that the diagnosis of rare diseases is more and more established by comprehensive genetic testing, rather than targeted investigations. Renal Fanconi syndrome (RFS) refers to a generalized dysfunction of the proximal tubule, leading to wasting of filtered solutes in the urine [2]. Clinical consequences include rickets and failure-to-thrive in children and osteomalacia in adults. RFS is very rare and can be acquired or inherited, with further subdivision of the latter based on the underlying genetics [3]. Here we describe a patient who went undiagnosed for more than 30 years despite numerous investigations. Her son presented with the same disorder in the neonatal period and routine genetic testing identified the specific cause, enabling the correct diagnosis also in the mother. Investigations previously carried out in the mother provide some clues to the underlying pathophysiology of the disorder.

## Case presentation

In March 1984, a 3-year old girl was admitted to Great Ormond Street Hospital (GOSH) for suspected RFS. She had been born at 35-weeks gestation and spent the first 2 weeks of life in hospital because of jaundice and recurrent hypoglycaemia. Investigations at the time included urine amino acid concentrations, which were considered consistent with a diagnosis of Hartnup disease, an autosomal dominant disorder caused by defective neutral amino acid re-uptake in the proximal tubule and which can be associated with a skin rash and neurological problems [4]. Over the next 2 years, she was noted to have delayed gross motor milestones, especially walking, thought to be secondary to the disorder and avoidance of sunlight was recommended and treatment with nicotinamide commenced. She developed bowing of her legs, which did not improve with cholecalciferol supplementation. At this point, her paediatrician suspected a diagnosis of RFS, rather than Hartnup disorder, commenced treatment with 1,25-OH cholecalciferol and referred her for further investigations. There was no family history of renal disease.

**Fig. 1:**
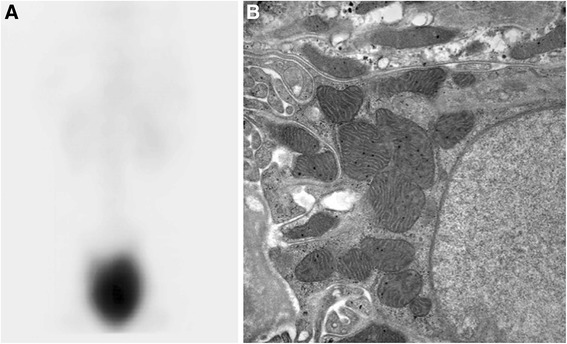
DMSA and biopsy of the kidneys. **a** DMSA scan showing tracer mostly in the bladder with no apparent uptake in the kidney, reflecting impaired proximal tubular transport of tracer in renal Fanconi syndrome. **b** Electron microscopy picture (magnification 19,500×) of a proximal tubular epithelial cell. Note the abnormal morphology of mitochondria, consistent with a mitochondrial cytopathy

On examination, she had genu varum and hepatomegaly, but no other dysmorphic findings. Her height was well below and weight on the 3rd percentile. Laboratory investigations revealed a metabolic acidosis, hypophosphataemia and markedly raised alkaline phosphatase (ALP: 2323 IU/l, normal for age < 280) with generalized aminoaciduria, glycosuria and tubular proteinuria, consistent with RFS. Leukocyte cystine content was normal and slit lamp examination did not show corneal cystine crystals, excluding a diagnosis of cystinosis, the most common cause of RFS in childhood. Radiological examination showed healing rickets with normal appearance of liver and kidneys on ultrasound. A diagnosis of autosomal recessive RFS was made and she received treatment with 1-OH cholecalciferol, phosphate, calcium and bicarbonate supplementation with clinical improvement of her rickets and good catch-up growth. Fasting glucose levels were repeatedly low. She was admitted again age 4 years for further investigations. A liver biopsy was normal, with no evidence for a glycogen storage disorder, tyrosinaemia or Wilson disease. A fructose–free diet had no effect, excluding fructose intolerance. Over the following years she was admitted multiple times for investigations, including 2 kidney biopsies (age 4 and 16 years), a bone marrow aspirate and serial measurements of glomerular filtration rate (GFR). Despite only moderately decreased GFR (47 ml/min/1.73m^2^), a DMSA scan of the kidneys age 5 years showed essentially no accumulation of tracer in the kidneys (Fig. 1a). A mild degree of nephrocalcinosis was noted first age 9 years, which remained stable thereafter.

She was transferred to adult services age 17 years with ongoing supplementation with 1-OH cholecalciferol, calcium, phosphate and bicarbonate. Another kidney biopsy was performed age 25 years, which now showed abnormal mitochondria (Fig. 1b). She reported episodes of fainting, especially if fasting for prolonged periods of time and consequently maintained a strictly regular dietary intake.

The patient had a first child age 34 years with normal pregnancy and postnatal examinations. She was counseled that due to the presumed autosomal recessive inheritance of her disorder, recurrence of the disease in her children would be unlikely.

Her second child was born at 30-weeks gestation with a weight of 1.53 kg after an uncomplicated pregnancy. He was noted to have recurrent hypoglycaemia in the neonatal period and transferred to GOSH. Investigations revealed hyperinsulinism, as well as highly raised ALP (2730 IU/l), glycosuria, tubular proteinuria, hypercalciuria and renal phosphate wasting, consistent with RFS. A screen of genes involved in hyperinsulinism revealed a heterozygous mutation c.187C > T; p.Arg63Trp in *HNF4A*, encoding Hepatocyte Nuclear factor 4A. The mutation was subsequently also confirmed in the mother. At last follow-up in December 2015 she remained clinically stable with ongoing biochemical features of RFS. Continuous glucose monitoring revealed prolonged overnight hypoglycaemia and post-prandial hyperglycaemia.

## Discussion and Conclusions

Our case highlights the dramatic recent improvements in our understanding of rare inherited disorders. The patient underwent multiple admissions and invasive investigations over a period of more than 20 years, yet without ever having a definitive diagnosis established, except for the initial misdiagnosis of Hartnup disorder. In contrast, her son was diagnosed within the first months of life, as part of our protocol for assessing persistent hypoglycaemia, which includes comprehensive genetic testing for genes involved in hyperinsulinism [5, 6]. Establishing the specific diagnosis is relevant, as it allows accurate genetic counseling, early identification of future children and optimized management of the hyperinsulinism with diazoxide to prevent episodes of hypoglycaemia.

Mutations in HNF4A were initially identified as a cause of diabetes (MODY type 1), but can also cause hyperinsulinism early in life [7]. The disorder is inherited in an autosomal dominant fashion. Notably, the additional finding of RFS is only associated with the specific mutation Arg63Trp (previously annotated as Arg76Trp) [8]. The cause for the renal involvement with this specific mutation is unclear, but the abnormal mitochondria on biopsy in our patient suggest an important role for HNF4A in maintaining mitochondrial integrity in the kidney. Not surprisingly, given the high energy demand of the proximal tubule, mitochondrial dysfunction is one of the best recognised causes of RFS [3]. Indeed, investigations in drosophila suggest an important role for HNF4 in the regulation of mitochondrial genes [9].

The recurrent hypoglycemia in our patient was noted, but erroneously attributed to the glycosuria by the investigating metabolic doctors at the time. It is important to note that loss of sugar in the urine, even with severe forms of familial renal gycosuria, where glucose loss can exceed 150 g per day, is not associated with hypoglycemia, presumably due to the tight regulation of blood glucose levels [10].

An interesting additional finding in our patient was the absence of renal DMSA accumulation. This is consistent with a previous report in RFS and likely reflects defective proximal tubular uptake of the tracer [11]. It is debated whether renal extraction of DMSA occurs via initial filtration and subsequent apical uptake or through basolateral uptake. The finding of absent renal extraction of DMSA, yet presence in the bladder clearly argues for apical uptake since impaired basolateral transport would prevent DMSA from appearing in the urine. Impaired luminal uptake is also consistent with experiments in megalin/cubilin deficient mice, which showed dependence of renal extraction on intact receptor-mediated endocytosis in the proximal tubule [12].

## Abbreviations

ALP: Alkaline phosphatase
DMSA: Dimercaptosuccinic acid
GFR: Glomerular filtration rate
GOSH: Great Ormond Street Hospital
MODY: Maturity Onset Diabetes in the Young
OMIM: Online Mendelian Inheritance in Man
RFS: Renal Fanconi Syndrome
